# Exploring the interaction between SNP genotype and postmenopausal hormone therapy effects on stroke risk

**DOI:** 10.1186/gm358

**Published:** 2012-07-13

**Authors:** Ying Huang, Dennis G Ballinger, Renee Stokowski, Erica Beilharz, Jennifer G Robinson, Simin Liu, Randal D Robinson, Victor W Henderson, Jacques E Rossouw, Ross L Prentice

**Affiliations:** 1 Public Health Sciences Division, Fred Hutchinson Cancer Research Center, 1100 Fairview Ave N, Seattle, WA 98109, USA; 2 Complete Genomics, Inc, 2071 Stierlin Court, Mountain View, CA 94043, USA; 3 Aria Diagnostics, 5945 Optical Court, San Jose, CA 95138, USA; 4 Departments of Epidemiology and Medicine, University of Iowa, 200 Medicine Admin. Bldg, Iowa City, IA 52242, USA; 5 School of Public Health, University of California at Los Angeles, 650 Charles E Young Dr. South, Los Angeles, CA 90095, USA; 6 School of Medicine, UT Heath Science Center, 7703 Floyd Curl Drive, San Antonio, TX 78229, USA; 7 Health Research & Policy (Epidemiology) and Neurology & Neurological Sciences, Stanford School of Medicine, 450 Serra Mall, Stanford, CA 94305, USA; 8 Women's Health Initiative Project Office, National Heart, Lung, and Blood Institute, 6701 Rockledge Drive, Bethesda, MD 20892, USA

## Abstract

**Background:**

Genome-wide association studies have identified several genomic regions that are associated with stroke risk, but these provide an explanation for only a small fraction of familial stroke aggregation. Genotype by environment interactions may contribute further to such an explanation. The Women's Health Initiative (WHI) clinical trial found increased stroke risk with postmenopausal hormone therapy (HT) and provides an efficient setting for evaluating genotype-HT interaction on stroke risk.

**Methods:**

We examined HT by genotype interactions for 392 SNPs selected from candidate gene studies, and 2,371 SNPs associated with changes in blood protein concentrations after hormone therapy, in analyses that included 2,045 postmenopausal women who developed stroke during WHI clinical trial and observational study follow-up and one-to-one matched controls. A two-stage procedure was implemented where SNPs passing the first stage screening based on marginal association with stroke risk were tested in the second stage for interaction with HT using case-only analysis.

**Results:**

The two-stage procedure identified two SNPs, rs2154299 and rs12194855, in the coagulation factor XIII subunit A (*F13A1*) region and two SNPs, rs630431 and rs560892, in the proprotein convertase subtilisin kexin 9 (*PCSK9*) region, with an estimated false discovery rate <0.05 based on interaction tests. Further analyses showed significant stroke risk interaction between these *F13A1 *SNPs and estrogen plus progestin (E+P) treatment for ischemic stroke and for ischemic and hemorrhagic stroke combined, and suggested interactions between *PCSK9 *SNPs with either E+P or estrogen-alone treatment.

**Conclusions:**

Genotype by environment interaction information may help to define genomic regions relevant to stroke risk. Two-stage analysis among postmenopausal women generates novel hypotheses concerning the *F13A1 *and *PCSK9 *genomic regions and the effects of hormonal exposures on postmenopausal stroke risk for subsequent independent validation.

## Background

In the past few years, genome-wide association studies have identified many common genetic variants associated with complex diseases. Most of the associations identified so far, however, explain only a small fraction of familial disease aggregation, which suggests that studies exploring additional human genome-related associations, such as those related to rare genetic variants, gene-gene interactions, and gene-environment interactions, could play an important role in accounting for the 'missing heritability' [1]. In the case of stroke, several genome-wide association studies have been performed to identify SNP associations, yet no single locus identified has been successfully replicated in a second study [2-9]. The complex nature of stroke risk, which may involve interactions between biological pathways, and the largely unexplained heritability suggest that gene-gene and gene-environment interactions could have a significant impact on the search for stroke-related SNPs [10,11].

A major challenge in assessing gene-environment interaction is the difficulty in characterizing environmental exposures precisely in an observational study setting, which has been the setting for most of the gene-environment interaction studies to date. Randomized controlled intervention trials, on the other hand, provide natural settings for incorporating gene-environment interaction assessment into the search for disease-susceptible SNPs since the treatment/intervention assignment is known exactly. An additional advantage of randomization is the resulting independence between intervention assignment and genotype, which justifies highly efficient case-only testing of interaction between SNPs and intervention in relation to disease risk [12-17].

The Women's Health Initiative (WHI) studies include two major components: a randomized controlled trial (CT) and an observational study (OS). The WHI trial included four randomized and controlled comparisons among postmenopausal women, in a partial factorial design [18,19]. Specifically, it comprised a postmenopausal hormone therapy component that involved two non-overlapping trials: an estrogen versus placebo (E-alone) trial among women who were post-hysterectomy, and an estrogen plus progestin versus placebo (E+P) trial among women with a uterus; a low-fat dietary modification (DM) versus usual diet component, and a calcium and vitamin D (CaD) supplementation versus placebo component.

The E+P trial was stopped early in 2002, triggered by an elevation of breast cancer risk and an overall unfavorable health benefit versus risk profile [20,21]. An important health risk was an approximate 40% elevation of stroke risk [22]. The E-alone trial was also stopped early, in 2004, primarily due to an elevation of stroke risk of similar magnitude to that seen for E+P [23,24]. The elevated stroke risks associated with the use of estrogen or estrogen plus progestin were not observed in the OS, where there was evidence of residual confounding [25]. Neither the DM nor the CaD trial yielded evidence of an intervention effect on stroke risk [26,27].

Here we study 2,763 candidate SNPs in relation to stroke incidence and hormone therapy (HT) intervention (E-alone and E+P) effects. These SNPs were evaluated in stroke cases and one-to-one matched controls in WHI cohorts as a 'core' WHI project that sought to identify SNPs that interact with hormone therapy effects on stroke risk.

Our analyses apply a novel two-stage procedure by first screening out unpromising SNPs based on marginal association tests with stroke using all available case-control samples from the WHI CT and OS, and then in the second stage investigating SNP-HT interaction only for SNPs meeting first-stage filtering criteria among stroke cases in the HT trials [28-30]. The independence between the test statistics for marginal association of SNPs and the case-only test statistics for interaction has been demonstrated in [29]. As a result, with a pre-determined threshold for marginal significance in the first-stage screening (chosen as 0.05), we only need to correct for the number of SNPs passing the first-stage filtering in evaluating gene-intervention interaction. This analytic approach preserves statistical power for interaction identification for the most promising SNPs.

## Materials and methods

### Study design and population

Enrollees in WHI trials were postmenopausal women aged 50 to 79 years who met component-specific eligibility criteria [31]. Women were randomized to a HT component, or a DM component, or both. At the one-year anniversary from enrollment participating women could be further randomized into the CaD supplementation component. A total of 68,132 women were enrolled into the trials between 1993 and 1998, among which there were 10,739 in E-alone, 16,608 in E+P, 48,835 in DM, and 36,282 in CaD components. The WHI OS enrolled 93,676 postmenopausal women and participants were followed for 6 to 10 years during the intervention phase of the WHI program. Details about distributions of demographic variables and stroke risk factors in the study cohort were published previously [31].

### Case and control selection

Overall, 2,096 stroke cases were considered for inclusion, including all stroke cases in the WHI clinical trials that developed between randomization and August 2007 and a subset of stroke cases in the OS that occurred following the time of case selection in a WHI pooled data genome-wide association study but prior to August 2007. Also considered were corresponding controls one-to-one matched to cases on baseline age, self-reported ethnicity, cohort (CT versus OS) and participation in each component if in the clinical trial, years since randomization/enrollment, baseline hysterectomy status, and prevalent stroke at enrollment. Among these, a total of 2,045 (97.6%) cases had adequate quantity and quality of DNA for this project. Of these, 149 (7.3%) cases had a prior history of stroke at baseline. Each case was matched to a qualifying control also having a suitable DNA specimen available, leading to an equal number of cases and controls in each CT or OS component. The number of included cases (or controls) was 351 in the E-alone trial, 438 in the E+P trial, 1,110 in the DM trial, 838 in the CaD trial (cases arising after CaD randomization only), and 373 in the OS (Table 1). The comparatively small number of cases from the OS occurs because of the exclusion of stroke cases included in the earlier WHI pooled DNA study mentioned above. Table 1 also presents the number of cases stratified further by stroke subcategory.

**Table 1:** Distribution of stroke cases and controls by type and CT/OS component

	E-alone	E+P	CaD	DM	CT	OS
Overall cases	351	438	838	1,110	1,672	373
Ischemic	263	319	575	695	1,110	226
Hemorrhagic	48	76	132	190	280	61
Other cases	40	43	131	225	282	86
Controls	351	438	838	1,110	1,672	373

CaD, calcium and vitamin D versus placebo supplementation; CT, randomized controlled trial; DM, low-fat dietary modification versus usual diet; E-alone, estrogen versus placebo; E+P, estrogen plus progestin versus placebo; OS, observational study.

Informed consent was obtained from each study participant and the research conformed to the Helsinki Declaration and to all pertinent local legislation.

### Laboratory methods and SNP selection

SNPs were genotyped and quality control criteria were applied at Perlegen Sciences (Mountain View, CA, USA). Genotyping and data cleaning methods have been described [32], with an average call rate of 99.8% and an average concordance rate of 99.8% for 157 blind duplicate samples.

Principal component analysis was used to characterize population structure and to identify genotyping artifacts. The top 20 principal components did not associate with common sources of experimental variability such as date of sample processing or hybridization performance for either chip design.

The 2,763 candidate SNPs evaluated in this paper cover 400 chromosomal regions with pairwise correlation r^2 ^between regions <0.2. Among these, 392 were selected from candidate genes previously reported to be related to stroke risk [4,33-35], and 2,371 were selected based on WHI studies of potential changes in blood protein concentrations following the initiation of hormone therapy [36,37].

### Statistical methods

A two-stage procedure as proposed in [29,30] was implemented for identification of SNP-HT interaction.

#### First stage filtering

In the first stage, the marginal effect for each SNP individually was estimated from a standard logistic regression of case (1) versus control (0) status on number of minor SNP alleles and potential confounding factors based on all case-control samples in CT and OS. The logistic regression model included smoking status, physical functioning score, history of treated diabetes, prevalent hypertension, current aspirin use and current statin use. Also included were the variables used for matching controls to cases in control selection and clinical trial randomization assignments. In addition, eigenvectors from the first ten principal components from correlation analysis of the genotype data were included to adjust for population stratification [38]. All SNPs with two-sided *P*-value < 0.05 were entered into the second stage for evaluation of interaction with HT.

#### Second stage

At the second stage, a two-component test statistic was used for each SNP to test for interaction with HT on stroke risk. The independence between HT treatment assignment and genotype in E-alone and E+P ensured by randomization allows the use of the case-only analysis for interaction testing, a more efficient method compared to standard case-control analysis. The two test statistic components are case-only tests for dependence of intervention odds ratios on SNP genotype for each of E-alone and E+P, respectively. These statistics arise as likelihood ratio test statistics in logistic regression of active (1) versus placebo randomization assignment on the number of minor SNP alleles with logistic regression location parameter offset by log q/(1 - q), where q is the fraction of women assigned to active intervention for the pertinent clinical trial component. That is, for cases within the E-alone or E+P trial separately, we fit a model with:

$$\text{logit Pr}(\text{Z}=1|\text{G})=\text{log}(\text{q}/(1-\text{q}))+\beta_{0}+\beta_{2}\text{G}$$

where Z = 0, 1 indicates assignment to placebo or treatment arm, respectively, and G is the number of minor alleles for the SNP considered.

The interaction tests for E-alone and E+P are independent of each other since they are based on non-overlapping sets of women. The two test statistics were added to yield a chi-square test with two degrees of freedom to test SNP interaction with HT on stroke risk for each of the SNPs. Control for multiple testing was carried out by requiring the estimated false discovery rate (FDR) [39] to be < 0.05.

SNPs of interest in the interaction test were subsequently examined for evidence of interaction effects with E-alone and E+P separately, both for all stroke cases and by major stroke subcategory (ischemic, hemorrhagic). Again, case-only analyses were employed, and for descriptive purposes, intervention odds ratios were estimated separately at zero, one, and two minor SNP alleles. Likelihood ratio tests with both one and two degrees of freedom (reflecting whether SNP genotype was modeled as a linear term in the number of minor SNP alleles, or a separate indicator variables for one and for two minor alleles) were examined to assess SNP by intervention interaction in these analyses. All significance levels (*P*-values) were two-sided. Note that the odds ratio estimates in these case-only analyses are (asymptotically) independent of those from the stage 1 analyses [29,30], so that only the number of SNPs included in stage 2 need to be considered in examining these odds ratios estimates for multiple testing-related biases.

The potential of SNP by HT interactions to contribute to the ability to discriminate between stroke cases and controls was evaluated by estimating areas under the receiver operating characteristic curves (AUC), and associated confidence intervals.

## Results

### Tests of interaction with hormonal therapy

Among the 2,763 SNPs studied, 112 SNPs passed the first stage screening threshold with a marginal effect *P*-value < 0.05, based on the additive allele log-odds model. Among these, 22 were from selected earlier literature reports and 90 were selected based on WHI proteomics studies. These 112 SNPs were then entered into the second stage analysis for testing of interaction with HT based on the simultaneous case-only test with two degrees of freedom. Information about the 112 SNPs is presented in Additional file 1.

Table 2 presents the top 10 SNPs ranked by *P*-value of the simultaneous test of interaction with HT, among the 112 SNPs entering the second stage analysis. Four SNPs had an interaction test FDR <0.05. Specifically, the top two SNPs, rs2154299 and rs12194855, ranked by interaction test *P*-value are located in an intronic region of the coagulation factor XIII subunit A gene (*F13A1*) on chromosome region 6p25. These SNPs were found to be associated with risk of ischemic stroke in an earlier study [35]. The two SNPs are in high linkage disequilibrium with each other, with pairwise correlation r^2 ^> 0.98, and have significant interaction with HT at the 0.05 level even after Bonferroni correction for the 112 interaction tests conducted in the second stage analysis. The other two SNPs identified are from an intronic region of the proprotein convertase subtilisin kexin 9 gene (*PCSK9*) in the genomic region of chromosome 1p32, the protein product of which was found to be increased by E-alone intervention in the WHI E-alone trial [36]. The two SNPs in the *PCSK9 *region are also in high linkage disequilibrium (r^2 ^> 0.98) with each other.

**Table 2:** Top ten SNPs identified by two-component test of interaction with E-alone or E+P

Rank	Rs#	Chr	Position	Allele	MAF	OR	Marginal association test *P*-value	HT interaction *P*-value	HT interaction FDR	E-alone interaction *P*-value	E+P interaction *P*-value	Gene
1	2154299	6	6231297	A/G	0.09	1.18	0.0299	0.00015	0.015	0.08111	0.00013	*F13A1*
2	12194855	6	6233241	G/A	0.09	1.19	0.0265	0.00026	0.015	0.10080	0.00020	*F13A1*
3	630431	1	55299911	G/A	0.34	0.91	0.0498	0.00068	0.025	0.01660	0.00291	*PCSK9*
4	568052	1	55297430	G/A	0.34	0.90	0.0450	0.00151	0.042	0.01939	0.00610	*PCSK9*
5	10028444	4	88654846	A/G	0.17	1.13	0.0482	0.03593	0.715	0.15318	0.03175	*SPARCL1*
6	1381633	4	88687876	G/A	0.23	1.12	0.0436	0.04131	0.715	0.05512	0.10068	*SPARCL1*
7	243842	16	54084923	C/T	0.39	0.87	0.0041	0.04671	0.715	0.02295	0.32839	*MMP2*
8	2817247	6	24580402	A/G	0.10	1.18	0.0227	0.05315	0.715	0.37301	0.02427	*GPLD1*
9	1982049	9	1.16E+08	T/G	0.36	1.10	0.0411	0.05747	0.715	0.03309	0.27896	*AMBP*
10	6413453	1	1.59E+08	A/G	0.11	0.86	0.0490	0.09972	0.881	0.78264	0.03322	*APOA2*

Rank, rank of SNPs based on HT interaction test with two degrees of freedom. Rs#, SNP identification (rs) number in dbSNP database. Chr, chromosome. Allele, minor/major allele. MAF, minor allele frequency in the study population. OR, odds ratio for increase of per minor allele. Marginal association test *P*-value, *P*-value based on test of SNP main effect assuming additive effect. HT interaction *P*-value: *P*-value based on two degrees of freedom joint test for interaction with HT. HT interaction FDR, FDR based on two degrees of freedom joint test for interaction with HT. E-alone interaction *P*-value, *P*-value based on test for interaction with E-alone. E+P interaction *P*-value, *P*-value based on test for interaction with E+P.

We further investigated these four SNPs for their interaction with the E-alone and E+P interventions separately and by stroke subcategory. Table 3 shows estimated HT intervention odds ratios and 95% confidence intervals as a function of the number of minor alleles for the two SNPs in the *F13A1 *region, for all stroke types together and for ischemic and hemorrhagic stroke separately. For both SNPs, a larger number of minor alleles (A for rs2154299 and G for rs12194855) appears to be associated with a lower E+P odds ratio. The pattern is consistent within ischemic or hemorrhagic stroke cases. The trends are in the same direction for E-alone, but not significant. Corresponding results for the two SNPs in the *PCSK9 *region are shown in Table 4. For both SNPs, there are suggestions that a larger number of minor alleles (G for rs630431 and G for rs568052) are associated with a lower E+P odds ratio, but a higher E-alone odds ratio, for all stroke types altogether and for ischemic stroke. The interactions of the two *PCSK9 *SNPs with either E-alone or E+P alone are only nominally significant.

**Table 3:** Stroke odds ratio for E-alone and E+P, by genotype of SNPs in *F13A1 *region

		SNP genotype							
				
	Number of cases	GG		GA		AA			
						
		OR	95% CI	OR	95% CI	OR	95% CI	p-2df	p-1df
**rs2154299**									
**E-alone**									
All	351	1.372	(1.086, 1.732)	0.920	(0.547, 1.548)	0.511	(0.094, 2.791)	0.21471	0.08111
Ischemic	263	1.614	(1.226, 2.124)	1.022	(0.566, 1.846)	0.682	(0.114, 4.079)	0.27045	0.10598
Hemorrhagic	48	0.756	(0.404, 1.414)	0.341	(0.069, 1.689)	NA	(NA, NA)	0.34339	0.34339
**E+P**									
All	438	1.517	(1.218, 1.889)	0.685	(0.459, 1.023)	0.238	(0.027, 2.13)	0.00066	0.00013
Ischemic	319	1.553	(1.198, 2.013)	0.68	(0.426, 1.087)	0.238	(0.027, 2.13)	0.00229	5.00E-04
Hemorrhagic	76	1.199	(0.724, 1.988)	0.635	(0.226, 1.784)	NA	(NA, NA)	0.27362	0.27362
									
**rs12194855**									
**E-alone**									
All	351	0.682	(0.114, 4.079)	0.890	(0.532, 1.492)	1.372	(1.086, 1.732)	0.25707	0.10080
Ischemic	263	0.682	(0.114, 4.079)	1.022	(0.566, 1.846)	1.614	(1.226, 2.124)	0.27045	0.10598
Hemorrhagic	48	NA	(NA, NA)	0.341	(0.069, 1.689)	0.756	(0.404, 1.414)	0.34339	0.34339
**E+P**									
All	438	0.238	(0.027, 2.130)	0.702	(0.471, 1.046)	1.509	(1.212, 1.88)	0.00097	2.00E-04
Ischemic	319	0.238	(0.027, 2.130)	0.703	(0.442, 1.118)	1.543	(1.190, 2.000)	0.00337	0.00076
Hemorrhagic	76	NA	(NA, NA)	0.635	(0.226, 1.784)	1.199	(0.724, 1.988)	0.27362	0.27362

OR, estimated intervention odds ratio. p-2df, *P*-value regressing randomization assignment on indicator for one or two minor alleles. p-1df, *P*-value regressing randomization assignment on number of minor alleles. NA indicates information (data) not available. CI, confidence interval.

**Table 4:** Stroke odds ratio for E-alone and E+P, by genotype of SNPs in *PCSK9 *region

		SNP genotype							
				
	Number of cases	AA		AG		GG			
						
		OR	95% CI	OR	95% CI	OR	95% CI	p-2df	p-1df
**rs630431**									
**E-alone**									
All	351	1.704	(1.227, 2.366)	1.064	(0.772, 1.468)	0.871	(0.499, 1.519)	0.04796	0.0166
Ischemic	263	1.994	(1.362, 2.919)	1.321	(0.906, 1.925)	0.767	(0.393, 1.498)	0.03845	0.01104
Hemorrhagic	48	0.651	(0.252, 1.678)	0.682	(0.306, 1.517)	0.682	(0.114, 4.079)	0.9971	0.94614
**E+P**									
All	438	0.935	(0.719, 1.217)	1.616	(1.192, 2.191)	1.978	(1.021, 3.834)	0.00919	0.00291
Ischemic	319	0.916	(0.667, 1.257)	1.550	(1.094, 2.197)	2.434	(1.126, 5.260)	0.01578	0.00401
Hemorrhagic	76	0.724	(0.399, 1.314)	2.143	(0.932, 4.929)	0.953	(0.192, 4.719)	0.10243	0.14652
									
**rs568052**									
**E-alone**									
All	351	1.716	(1.233, 2.389)	1.050	(0.762, 1.446)	0.909	(0.524, 1.575)	0.04909	0.01939
Ischemic	263	2.019	(1.373, 2.967)	1.294	(0.889, 1.882)	0.818	(0.424, 1.578)	0.04487	0.01272
Hemorrhagic	48	0.651	(0.252, 1.678)	0.682	(0.306, 1.517)	0.682	(0.114, 4.079)	0.9971	0.94614
**E+P**									
All	438	0.961	(0.738, 1.251)	1.555	(1.150, 2.103)	1.978	(1.021, 3.834)	0.02051	0.0061
Ischemic	319	0.953	(0.693, 1.309)	1.474	(1.043, 2.082)	2.434	(1.126, 5.260)	0.03335	0.00918
Hemorrhagic	76	0.724	(0.399, 1.314)	2.143	(0.932, 4.929)	0.953	(0.192, 4.719)	0.10243	0.14652

OR, estimated intervention odds ratio. p-2df, *P*-value regressing randomization assignment on indicator for one or two minor alleles. p-1df, *P*-value regressing randomization assignment on number of minor alleles. CI, confidence interval.

The majority (81%) of the case-control samples are from European-ancestry women. In Additional files 2 and 3 we provide *P*-values for interaction between HT component and the four SNPs in the *F13A1 and PCSK9 *regions, and the estimated intervention odds ratios and 95% confidence intervals as a function of the number of minor alleles, among women of European ancestry specifically. The patterns that we observe are quite similar to the overall patterns.

Additionally, we examined the joint effect of the *F13A1 *SNPs and *PCSK9 *SNPs in interacting with E+P intervention, using case-only analysis. Based on logistic regression applied to cases in the E+P trial, where the indicator for active treatment is regressed on genotypes of rs2154299 and rs630431 together, both SNPs showed nominally significant interactions. Specifically, when the indicators for one or two minor alleles are included in the regression, the nominal *P*-values based on likelihood ratio test with two degrees of freedom for rs2154299 and rs630431 were 0.0009 and 0.013 when all stroke types are considered and 0.004 and 0.025 for ischemic stroke. When numbers of minor alleles are included in the regression assuming 'monotone' interaction, the nominal *P*-values based on a likelihood ratio test with one degree of freedom for rs2154299 and rs630431 were 0.0002 and 0.005 when all stroke types were considered and 0.001 and 0.008 for ischemic stroke.

To examine the added benefit of including SNPs in the two regions for discriminating stroke cases from controls, we calculated the AUC from logistic regression analyses that included matching covariates, clinical trial randomization assignments for each of the four interventions, and the potential confounding factors listed above. This gave an AUC (95% confidence interval) of 0.645 (0.634, 0.666) for overall stroke. When rs2154299 and rs630431 were further incorporated into the risk model including main effect indicator variables for one and two minor alleles and corresponding HT intervention interaction indicator variables, the AUC increased to only 0.651 (0.642, 0.675). A bootstrap test of significance for the increase in AUC gave a nominal *P*-value of 0.045.

## Discussion

In this report we evaluated the interaction between hormone therapy and 2,763 SNPs on stroke incidence in the WHI clinical trial, through the use of a two-stage testing procedure that conducts interaction tests among only the SNPs passing a first stage filtering based on a marginal association test with stroke incidence. With gene-environment interaction being the major focus here, this type of two-stage procedure allows multiple-testing adjustment to be restricted to a smaller set of SNPs passing the first stage screening. This separate adjustment of interaction testing from main SNP effect testing is justified by the independence between the two test statistics [29], and provides a powerful alternative to the procedure of applying interaction testing to all SNPs. Note, however, that this testing procedure would not be sensitive for interaction detection for SNPs for which there is little evidence for a marginal association with stroke risk.

Our study is novel in being nested within the randomized controlled WHI clinical trial, implying randomization assignments that are known and statistically independent of genotype. This independence allows case-only interaction tests with their substantial efficiency gains compared to case-control interaction tests.

Our analyses identified interesting SNPs on two genomic regions: rs2154299 and rs12194855 from the *F13A1 *region of chromosome 6 with a strong interaction with E+P; and rs630431 and rs568052 from the *PCSK9 *region of chromosome 1 with suggestive evidence for an interaction with HT. The *F13A1 *gene encodes subunit A of coagulation factor XIII, which in its active form is involved in the blood coagulation process. Upon activation by thrombin, factor XIIIa acts on fibrin to form cross-links between fibrin molecules to form an insoluble clot. Previous analyses of the WHI trial data have shown that ischemic stroke risk is directly associated with baseline D-dimer levels and that HT increases D-dimer levels [40]. D-dimer is a fibrin degradation product containing two cross-linked D fragments of the fibrinogen protein, which is present in the blood after a blood clot is degraded by fibrinolysis. Previously, SNPs in the coding region of *F13A1 *(rs5985 for Val34Leu and rs3024477 for Tyr204Phe) were reported to be associated with risk of ischemic stroke [4]. However, neither of these SNPs passed the first stage screening in our study, and both are in very low linkage disequilibrium with rs2154299 and rs12194855 (r^2 ^< 0.01).

Variations in the *PCSK9 *gene have been shown to be related to the risk of large-vessel atherosclerosis stroke [41]. *PCSK9 *encodes the proprotein convertase subtilisin/kexin type 9 protein, an important regulator of plasma low-density lipoprotein cholesterol. PCSK9 protein binds to and degrades the low density lipoprotein cholesterol receptor. *PCSK9 *loss-of-function mutations result in low levels of low density lipoprotein cholesterol and protect against coronary heart disease while gain-of-function mutations have the opposite effect [42]. The possibility of using PCSK9 in the treatment of hypercholesterolemia has fueled considerable research into related molecular mechanisms [43]. In the WHI hormone trials, higher levels of baseline low-density lipoprotein cholesterol were related to the risk of ischemic stroke, and interacted with E-alone to increase risk [40]. Moreover, in the WHI E-alone trial, blood PCSK9 protein levels were observed to increase with E-alone intervention [36]. These findings, together with the significant interactions discovered in this study between *PCSK9 *and both E-alone and E+P, makes *PCSK9 *another interesting candidate for future study of a relationship with hormone therapy and stroke risk and suggests plausible actions through blood low density lipoprotein cholesterol levels.

In our analysis, interactions of SNPs from the two regions with hormone therapy led to only a very small increase in the estimated ability to distinguish stroke cases from controls. Note that AUCs reported here represent classification accuracy of the models in the matched case-control sample and are of exploratory nature only. On the one hand, matching by factors such as age and prevalent stroke in the case-control sample makes the estimated AUC a somewhat distorted, and likely reduced, estimate of the population AUC; on the other hand, even the small increase in estimated AUC when interacting SNPs are added may be optimistic since these SNPs were identified in the same dataset used to estimate AUC.

When the simultaneous interaction test with E-alone and E+P is further separated into its constituents, we observe highly significant evidence of interaction between the two *F13A1 *SNPs with E+P and nominally significant evidence of interactions between the two *PCSK9 *SNPs with E+P and E-alone for total stroke and for ischemic stroke specifically. The number of hemorrhagic stroke events is too small for any clear conclusions. The results suggest that among women with identified SNPs in the *F13A1 *region (GG homozygotes for rs2154299 and AA homozygotes for rs12104885), E-alone or E+P increased risk of ischemic stroke by about half, with little evidence of an HT effect on stroke risk if one or more minor SNP alleles are present. Within the *PCSK9 *region, among women with the AA genotype for SNPs rs630431 or rs568052 the risk of ischemic stroke was approximately doubled by E-alone; in contrast, risk elevations of a similar magnitude by E+P were noted among women with the GG genotype. Considering our procedure of selection based on most significant interactions, the magnitude of HR odds ratio variations between genotypes observed in this project is subject to 'winner's curse' and could be exaggerated due to the fact that 112 SNPs were considered in the second stage analyses. These analyses represent an early step in assessing the role of gene-environment interactions to help explain familial stroke patterns. The analyses also generate interesting hypotheses concerning genotype interactions with hormone therapy in relation to stroke risk that require further confirmatory study.

## Conclusions

Two-stage analysis among postmenopausal women generates novel hypotheses about the interaction between *F13A1 *and *PCSK9 *genomic regions and the effects of hormonal exposures on postmenopausal stroke risk for subsequent independent validation. These analyses represent an early step in assessing the role of genotype by environment interaction to help explain familial stroke aggregation.

## Abbreviations

AUC: area under the receiver operating characteristic curve; CaD trial: calcium and vitamin D versus placebo supplementation component; CT: randomized controlled trial; DM trial: low-fat dietary modification versus usual diet component; E-alone trial: estrogen versus placebo; E+P trial: estrogen plus progestin versus placebo; *F13A1*: coagulation factor XIII subunit A; FDR: false discovery rate; HT: hormone therapy; OS: observational study; *PCSK9*: proprotein convertase subtilisin kexin 9; SNP: single nucleotide polymorphism; WHI: Women's Health Initiative.

## Competing interests

The authors declare that they have no competing interests.

## Authors' contributions

All authors were involved in development and/or critical review and revision of the manuscript. Additionally, DB, RS, and EB had primary responsibility for project genotyping; YH and RP had primary responsibility for data analysis; JR and RP had responsibility for clinical data; and DB and RP had primary administrative responsibility for this research project. All authors have read and approved the manuscript for publication.

## Supplementary Material

Additional file 1 **The 112 SNPs tested in stage 2, ranked by significance of two-component test of interaction with E-alone or E+P**. This file extends the information provided in Table 2 for the 10 SNPs that were top-ranked according to hormone therapy interaction with stroke risk to the entire set of 112 SNPs having a marginal association nominal significance level <0.05. Click here for file

Additional file 2 **Stroke odds ratio for E-alone and E+P, by genotype of SNPs in the *F13A1 region *in women of European ancestry**. This file presents analyses corresponding to Table 3, with cases and controls restricted to be of European ancestry. Click here for file

Additional file 3 **Stroke odds ratio for E-alone and E+P, by genotype of SNPs in *PCSK9 region *for women with European ancestry**. This file presents analyses corresponding to Table 4, with cases and controls restricted to be of European ancestry. Click here for file
