# Supplementary material for: Exploring the interaction between SNP genotype and postmenopausal hormone therapy effects on stroke risk
Source: Genome Med. 2012 Jul 13;4(7):57. doi: 10.1186/gm358 (PMC3580413; doi:10.1186/gm358)
Supplement: Additional file 1 — The 112 SNPs tested in stage 2, ranked by significance of two-component test of interaction with E-alone or E+P. This file extends the information provided in Table 2 for the 10 SNPs that were top-ranked according to hormone therapy interaction with stroke risk to the entire set of 112 SNPs having a marginal association nominal significance level <0.05. [file gm358-S1.DOC]

**Additional File 1.** The 112 SNPs tested in stage 2, ranked by significance of two-component test of interaction with E-alone or E+P.

| rank^‡^ | Rs#* | Chr | Position | Allele* | MAF* | OR* | Marginal Association Test *P*-value^‡^ | HT Interaction P-value^‡^ | HT Interaction FDR^‡^ | E-alone Interaction P-value^‡^ | E+P Interaction P-value^‡^ | Gene |
| --- | --- | --- | --- | --- | --- | --- | --- | --- | --- | --- | --- | --- |
| 1 | 2154299 | 6 | 6231297 | A/G | 0.09 | 1.18 | 0.0299 | 0.00015 | 0.015 | 0.08111 | 0.00013 | F13A1 |
| 2 | 12194855 | 6 | 6233241 | G/A | 0.09 | 1.19 | 0.0265 | 0.00026 | 0.015 | 0.10080 | 0.00020 | F13A1 |
| 3 | 630431 | 1 | 55299911 | G/A | 0.34 | 0.91 | 0.0498 | 0.00068 | 0.025 | 0.01660 | 0.00291 | PCSK9 |
| 4 | 568052 | 1 | 55297430 | G/A | 0.34 | 0.90 | 0.0450 | 0.00151 | 0.042 | 0.01939 | 0.00610 | PCSK9 |
| 5 | 10028444 | 4 | 88654846 | A/G | 0.17 | 1.13 | 0.0482 | 0.03593 | 0.715 | 0.15318 | 0.03175 | SPARCL1 |
| 6 | 1381633 | 4 | 88687876 | G/A | 0.23 | 1.12 | 0.0436 | 0.04131 | 0.715 | 0.05512 | 0.10068 | SPARCL1 |
| 7 | 243842 | 16 | 54084923 | C/T | 0.39 | 0.87 | 0.0041 | 0.04671 | 0.715 | 0.02295 | 0.32839 | MMP2 |
| 8 | 2817247 | 6 | 24580402 | A/G | 0.10 | 1.18 | 0.0227 | 0.05315 | 0.715 | 0.37301 | 0.02427 | GPLD1 |
| 9 | 1982049 | 9 | 115882466 | T/G | 0.36 | 1.10 | 0.0411 | 0.05747 | 0.715 | 0.03309 | 0.27896 | AMBP |
| 10 | 6413453 | 1 | 159458940 | A/G | 0.11 | 0.86 | 0.0490 | 0.09972 | 0.881 | 0.78264 | 0.03322 | APOA2 |
| 11 | 243845 | 16 | 54083988 | A/G | 0.40 | 0.88 | 0.0087 | 0.10327 | 0.881 | 0.05808 | 0.32984 | MMP2 |
| 12 | 17012853 | 4 | 88673208 | T/C | 0.20 | 1.13 | 0.0290 | 0.11290 | 0.881 | 0.28483 | 0.07280 | SPARCL1 |
| 13 | 103294 | 19 | 59489660 | T/C | 0.19 | 1.12 | 0.0423 | 0.11645 | 0.881 | 0.03862 | 0.87911 | LILRA3 |
| 14 | 1329423 | 1 | 194913010 | C/T | 0.24 | 1.13 | 0.0255 | 0.12522 | 0.881 | 0.48215 | 0.05569 | CFH |
| 15 | 1934074 | 1 | 158164012 | T/C | 0.36 | 1.14 | 0.0076 | 0.14524 | 0.881 | 0.05265 | 0.74733 | TAGLN2 |
| 16 | 2512673 | 11 | 82838981 | T/G | 0.32 | 0.87 | 0.0070 | 0.14802 | 0.881 | 0.06137 | 0.57115 | DLG2 |
| 17 | 9863056 | 3 | 351457 | A/T | 0.14 | 1.14 | 0.0446 | 0.15641 | 0.881 | 0.12328 | 0.24786 | CHL1 |
| 18 | 2512676 | 11 | 82844368 | G/T | 0.36 | 0.89 | 0.0200 | 0.15927 | 0.881 | 0.05944 | 0.72755 | DLG2 |
| 19 | 3753394 | 1 | 194887540 | T/C | 0.26 | 1.11 | 0.0386 | 0.16188 | 0.881 | 0.78383 | 0.05896 | CFH |
| 20 | 2283508 | 16 | 16180009 | A/G | 0.48 | 1.14 | 0.0055 | 0.16804 | 0.881 | 0.08290 | 0.45427 | ABCC6 |
| 21 | 6018 | 1 | 167778502 | G/T | 0.06 | 0.82 | 0.0461 | 0.17456 | 0.881 | 0.06737 | 0.70340 | F5 |
| 22 | 9332730 | 6 | 32019988 | C/G | 0.04 | 1.26 | 0.0406 | 0.18898 | 0.881 | 0.09425 | 0.46580 | CFB |
| 23 | 243849 | 16 | 54081206 | T/C | 0.16 | 1.17 | 0.0112 | 0.20328 | 0.881 | 0.13564 | 0.32729 | MMP2 |
| 24 | 163913 | 19 | 6673635 | C/T | 0.19 | 1.14 | 0.0260 | 0.21101 | 0.881 | 0.16556 | 0.27552 | C3 |
| 25 | 13009405 | 2 | 101375833 | A/C | 0.49 | 0.91 | 0.0397 | 0.21600 | 0.881 | 0.08104 | 0.88442 | CREG2 |
| 26 | 10503814 | 8 | 27510492 | T/C | 0.04 | 1.41 | 0.0009 | 0.22727 | 0.881 | 0.09063 | 0.75182 | CLU |
| 27 | 10878397 | 12 | 64962700 | G/A | 0.47 | 0.90 | 0.0217 | 0.25332 | 0.881 | 0.57628 | 0.11873 | HELB |
| 28 | 2267951 | 6 | 133061551 | T/C | 0.31 | 1.10 | 0.0430 | 0.26983 | 0.881 | 0.62101 | 0.12326 | VNN1 |
| 29 | 7896471 | 10 | 101778298 | T/G | 0.04 | 0.76 | 0.0201 | 0.27526 | 0.881 | 0.13061 | 0.58710 | CPN1 |
| 30 | 17186372 | 1 | 120302474 | G/C | 0.06 | 0.81 | 0.0442 | 0.27796 | 0.881 | 0.32341 | 0.20798 | NOTCH2 |
| 31 | 17258599 | 1 | 120316807 | C/T | 0.06 | 0.81 | 0.0415 | 0.27796 | 0.881 | 0.32341 | 0.20798 | NOTCH2 |
| 32 | 12139175 | 1 | 158169410 | G/C | 0.31 | 1.13 | 0.0141 | 0.28171 | 0.881 | 0.11153 | 0.97108 | TAGLN2 |
| 33 | 4974538 | 3 | 195543601 | T/C | 0.12 | 0.85 | 0.0128 | 0.28611 | 0.881 | 0.21837 | 0.32030 | CPN2 |
| 34 | 3761008 | 19 | 801023 | C/T | 0.15 | 1.17 | 0.0152 | 0.29015 | 0.881 | 0.17837 | 0.41531 | CFD |
| 35 | 1129770 | 5 | 79122639 | A/G | 0.16 | 1.17 | 0.0113 | 0.29488 | 0.881 | 0.14443 | 0.57628 | CMYA5 |
| 36 | 2930902 | 19 | 838128 | G/A | 0.15 | 1.16 | 0.0169 | 0.29527 | 0.881 | 0.18987 | 0.39579 | CFD |
| 37 | 6033 | 1 | 167788477 | G/A | 0.08 | 0.82 | 0.0167 | 0.30023 | 0.881 | 0.12162 | 0.92028 | F5 |
| 38 | 17057444 | 8 | 27509710 | G/C | 0.04 | 1.28 | 0.0262 | 0.30520 | 0.881 | 0.12452 | 0.90563 | CLU |
| 39 | 6036 | 1 | 167782350 | T/C | 0.07 | 0.78 | 0.0056 | 0.31103 | 0.881 | 0.14391 | 0.65466 | F5 |
| 40 | 3826946 | 19 | 801199 | A/T | 0.16 | 1.18 | 0.0087 | 0.32853 | 0.881 | 0.14302 | 0.77582 | CFD |
| 41 | 6015 | 1 | 167786518 | A/G | 0.07 | 0.78 | 0.0061 | 0.32979 | 0.881 | 0.14391 | 0.77338 | F5 |
| 42 | 6027 | 1 | 167750185 | C/T | 0.06 | 0.80 | 0.0247 | 0.33257 | 0.881 | 0.14369 | 0.80057 | F5 |
| 43 | 12640408 | 4 | 88658130 | A/G | 0.19 | 1.12 | 0.0489 | 0.33837 | 0.881 | 0.51846 | 0.18584 | SPARCL1 |
| 44 | 11097162 | 4 | 88671932 | T/C | 0.43 | 1.10 | 0.0336 | 0.43472 | 0.978 | 0.79256 | 0.20634 | SPARCL1 |
| 45 | 2093785 | 9 | 115890897 | A/G | 0.30 | 1.11 | 0.0369 | 0.44619 | 0.978 | 0.25252 | 0.58094 | AMBP |
| 46 | 6710535 | 2 | 127907430 | G/C | 0.36 | 0.91 | 0.0477 | 0.44837 | 0.978 | 0.46816 | 0.29916 | PROC |
| 47 | 3821181 | 2 | 187977176 | G/C | 0.18 | 0.87 | 0.0301 | 0.45311 | 0.978 | 0.32249 | 0.43689 | TFPI |
| 48 | 212075 | 16 | 16186784 | G/A | 0.47 | 0.91 | 0.0473 | 0.45644 | 0.978 | 0.39344 | 0.35930 | ABCC6 |
| 49 | 6753288 | 2 | 127886369 | A/G | 0.44 | 1.10 | 0.0431 | 0.46549 | 0.978 | 0.87208 | 0.22015 | PROC |
| 50 | 150467 | 16 | 16186549 | G/C | 0.46 | 0.91 | 0.0490 | 0.48581 | 0.978 | 0.37739 | 0.41491 | ABCC6 |
| 51 | 3211752 | 13 | 112835460 | G/A | 0.46 | 1.10 | 0.0492 | 0.50319 | 0.978 | 0.32584 | 0.52288 | F10 |
| 52 | 10469833 | 2 | 3689923 | G/T | 0.06 | 1.19 | 0.0428 | 0.50407 | 0.978 | 0.24693 | 0.86363 | COLEC11 |
| 53 | 13084688 | 3 | 217241 | G/A | 0.47 | 1.12 | 0.0313 | 0.50783 | 0.978 | 0.24658 | 0.91055 | CHL1 |
| 54 | 1878200 | 2 | 189544923 | T/A | 0.45 | 0.91 | 0.0359 | 0.51473 | 0.978 | 0.38439 | 0.44962 | COL3A1 |
| 55 | 1031796 | 4 | 88670603 | C/A | 0.46 | 1.10 | 0.0379 | 0.54356 | 0.978 | 0.95806 | 0.27006 | SPARCL1 |
| 56 | 2298830 | 11 | 83495008 | T/C | 0.16 | 0.80 | 0.0303 | 0.54621 | 0.978 | 0.41441 | 0.46103 | DLG2 |
| 57 | 6769789 | 3 | 52849486 | C/T | 0.43 | 1.14 | 0.0219 | 0.54875 | 0.978 | 0.40100 | 0.48176 | ITIH4 |
| 58 | 12498514 | 4 | 167021567 | C/T | 0.30 | 1.11 | 0.0332 | 0.54898 | 0.978 | 0.28515 | 0.81130 | TLL1 |
| 59 | 11602775 | 11 | 83722544 | T/G | 0.13 | 1.23 | 0.0045 | 0.58311 | 0.978 | 0.35130 | 0.64676 | DLG2 |
| 60 | 1799810 | 2 | 127892510 | T/A | 0.42 | 1.11 | 0.0259 | 0.59064 | 0.978 | 0.47089 | 0.46526 | PROC |
| 61 | 10792722 | 11 | 83499829 | G/A | 0.16 | 0.76 | 0.0129 | 0.60939 | 0.978 | 0.98027 | 0.31974 | DLG2 |
| 62 | 9389025 | 6 | 133046684 | A/T | 0.23 | 1.12 | 0.0428 | 0.61867 | 0.978 | 0.98278 | 0.32721 | VNN1 |
| 63 | 963259 | 11 | 83621770 | C/A | 0.27 | 1.21 | 0.0302 | 0.61936 | 0.978 | 0.38115 | 0.66196 | DLG2 |
| 64 | 7698450 | 4 | 167046988 | C/T | 0.29 | 1.11 | 0.0324 | 0.64224 | 0.978 | 0.70897 | 0.38766 | TLL1 |
| 65 | 2817205 | 6 | 24585113 | A/G | 0.47 | 0.91 | 0.0498 | 0.64570 | 0.978 | 0.36192 | 0.83457 | GPLD1 |
| 66 | 243839 | 16 | 54086912 | G/A | 0.22 | 1.13 | 0.0312 | 0.66303 | 0.978 | 0.36534 | 0.96091 | MMP2 |
| 67 | 6938367 | 6 | 133067395 | T/G | 0.32 | 0.82 | 0.0001 | 0.66944 | 0.978 | 0.60771 | 0.46280 | VNN1 |
| 68 | 12931472 | 16 | 16188508 | G/A | 0.47 | 1.14 | 0.0053 | 0.67922 | 0.978 | 0.52407 | 0.54423 | ABCC6 |
| 69 | 6441989 | 3 | 46449903 | A/G | 0.44 | 1.12 | 0.0086 | 0.68852 | 0.978 | 0.47125 | 0.63348 | LTF |
| 70 | 9341105 | 2 | 217209064 | G/A | 0.27 | 0.90 | 0.0352 | 0.69091 | 0.978 | 0.39816 | 0.87286 | IGFBP2 |
| 71 | 6790273 | 3 | 195494 | G/A | 0.22 | 0.88 | 0.0394 | 0.69275 | 0.978 | 0.97388 | 0.39188 | CHL1 |
| 72 | 2930894 | 19 | 832161 | A/G | 0.28 | 1.19 | 0.0006 | 0.70721 | 0.978 | 0.62674 | 0.49934 | CFD |
| 73 | 6428370 | 1 | 195111216 | G/A | 0.33 | 1.13 | 0.0433 | 0.71012 | 0.978 | 0.41229 | 0.91122 | CFHR1 |
| 74 | 2373249 | 3 | 46445732 | C/G | 0.37 | 0.89 | 0.0112 | 0.72002 | 0.978 | 0.42596 | 0.87908 | LTF |
| 75 | 9637646 | 4 | 167022978 | T/C | 0.34 | 1.11 | 0.0362 | 0.72609 | 0.978 | 0.79391 | 0.44950 | TLL1 |
| 76 | 12711953 | 2 | 3678897 | A/G | 0.06 | 1.24 | 0.0223 | 0.74111 | 0.978 | 0.47682 | 0.76027 | COLEC11 |
| 77 | 17300770 | 6 | 24570999 | C/G | 0.13 | 0.85 | 0.0187 | 0.74268 | 0.978 | 0.83241 | 0.45823 | GPLD1 |
| 78 | 344540 | 19 | 6638768 | A/G | 0.39 | 1.10 | 0.0428 | 0.75535 | 0.978 | 0.93970 | 0.45611 | C3 |
| 79 | 5981 | 6 | 6167381 | G/T | 0.17 | 1.14 | 0.0321 | 0.75874 | 0.978 | 0.76784 | 0.49527 | F13A1 |
| 80 | 958480 | 9 | 5719377 | A/T | 0.46 | 0.91 | 0.0465 | 0.77133 | 0.978 | 0.47197 | 0.96510 | KIAA1432 |
| 81 | 292009 | 1 | 22863295 | G/A | 0.15 | 1.17 | 0.0124 | 0.77766 | 0.978 | 0.48151 | 0.93103 | C1QB |
| 82 | 12500750 | 4 | 167033008 | G/C | 0.25 | 1.12 | 0.0355 | 0.78716 | 0.978 | 0.65447 | 0.59779 | TLL1 |
| 83 | 212084 | 16 | 16136708 | T/C | 0.40 | 0.90 | 0.0276 | 0.79652 | 0.978 | 0.53759 | 0.78422 | ABCC6 |
| 84 | 6035 | 1 | 167788473 | C/T | 0.08 | 1.21 | 0.0125 | 0.80803 | 0.978 | 0.69883 | 0.59892 | F5 |
| 85 | 3829793 | 1 | 159461024 | G/C | 0.33 | 0.90 | 0.0294 | 0.81620 | 0.978 | 0.91882 | 0.52926 | APOA2 |
| 86 | 1683565 | 19 | 831602 | G/A | 0.28 | 1.19 | 0.0006 | 0.81870 | 0.978 | 0.64026 | 0.66994 | CFD |
| 87 | 2071044 | 3 | 52822641 | T/C | 0.50 | 1.19 | 0.0045 | 0.84180 | 0.978 | 0.73602 | 0.63095 | ITIH1 |
| 88 | 17376089 | 6 | 6145359 | A/G | 0.14 | 1.17 | 0.0137 | 0.84371 | 0.978 | 0.66021 | 0.70178 | F13A1 |
| 89 | 16988566 | 20 | 3395224 | G/A | 0.10 | 0.84 | 0.0385 | 0.84395 | 0.978 | 0.81165 | 0.59505 | ATRN |
| 90 | 3792640 | 5 | 40968595 | A/G | 0.18 | 1.14 | 0.0357 | 0.84972 | 0.978 | 0.85049 | 0.59011 | C7 |
| 91 | 10516852 | 4 | 91062673 | A/T | 0.11 | 0.86 | 0.0422 | 0.84993 | 0.978 | 0.81189 | 0.60429 | MMRN1 |
| 92 | 4685448 | 3 | 227344 | A/G | 0.39 | 1.14 | 0.0303 | 0.85459 | 0.978 | 0.60353 | 0.83279 | CHL1 |
| 93 | 16889242 | 6 | 24538725 | C/A | 0.14 | 0.87 | 0.0442 | 0.85469 | 0.978 | 0.58328 | 0.90899 | GPLD1 |
| 94 | 2073495 | 3 | 46455962 | G/C | 0.31 | 0.91 | 0.0492 | 0.85952 | 0.978 | 0.96666 | 0.58324 | LTF |
| 95 | 2649663 | 11 | 57113881 | G/A | 0.20 | 1.13 | 0.0358 | 0.85979 | 0.978 | 0.74954 | 0.65454 | SERPING1 |
| 96 | 9809528 | 3 | 225758 | G/A | 0.39 | 1.15 | 0.0245 | 0.86144 | 0.978 | 0.65000 | 0.76116 | CHL1 |
| 97 | 17405909 | 5 | 79141263 | A/G | 0.13 | 1.19 | 0.0088 | 0.87750 | 0.978 | 0.60940 | 0.98587 | CMYA5 |
| 98 | 17147136 | 11 | 83673251 | C/T | 0.09 | 1.21 | 0.0479 | 0.89227 | 0.978 | 0.98499 | 0.63329 | DLG2 |
| 99 | 17147160 | 11 | 83682972 | G/T | 0.15 | 1.18 | 0.0319 | 0.89457 | 0.978 | 0.78233 | 0.70192 | DLG2 |
| 100 | 1846568 | 11 | 57114290 | T/G | 0.20 | 1.14 | 0.0197 | 0.90827 | 0.978 | 0.96642 | 0.66237 | SERPING1 |
| 101 | 2745434 | 6 | 133057369 | T/A | 0.34 | 0.86 | 0.0037 | 0.91142 | 0.978 | 0.67400 | 0.92631 | VNN1 |
| 102 | 2839628 | 21 | 43325937 | G/C | 0.31 | 0.89 | 0.0262 | 0.91591 | 0.978 | 0.68757 | 0.90597 | CBS |
| 103 | 7343 | 5 | 79131477 | A/G | 0.13 | 1.15 | 0.0376 | 0.92228 | 0.978 | 0.86002 | 0.71771 | CMYA5 |
| 104 | 1515089 | 11 | 83656690 | T/C | 0.19 | 1.22 | 0.0056 | 0.92306 | 0.978 | 0.90269 | 0.70319 | DLG2 |
| 105 | 1515090 | 11 | 83725082 | A/C | 0.14 | 1.18 | 0.0209 | 0.92967 | 0.978 | 0.73022 | 0.86961 | DLG2 |
| 106 | 9467176 | 6 | 24572886 | A/G | 0.17 | 0.86 | 0.0151 | 0.93305 | 0.978 | 0.86741 | 0.73933 | GPLD1 |
| 107 | 4944478 | 11 | 83684158 | C/T | 0.19 | 1.21 | 0.0091 | 0.93785 | 0.978 | 0.88745 | 0.74208 | DLG2 |
| 108 | 6441984 | 3 | 46445090 | A/T | 0.48 | 1.13 | 0.0088 | 0.94295 | 0.978 | 0.77138 | 0.85576 | LTF |
| 109 | 8128028 | 21 | 43339612 | T/C | 0.33 | 0.90 | 0.0270 | 0.96572 | 0.988 | 0.94190 | 0.79960 | CBS |
| 110 | 17323806 | X | 100825720 | A/G | 0.08 | 1.20 | 0.0192 | 0.97703 | 0.988 | 0.95243 | 0.83588 | GLA |
| 111 | 7101982 | 11 | 84134088 | C/G | 0.44 | 1.15 | 0.0464 | 0.97914 | 0.988 | 0.83731 | 0.99829 | DLG2 |
| 112 | 3792633 | 5 | 40965153 | C/G | 0.14 | 0.87 | 0.0398 | 0.99936 | 0.999 | 0.98951 | 0.97338 | C7 |

*Rs# – SNP identification (rs) number in dbSNP database; *Allele – Minor/Major Allele; *MAF -- Minor allele frequency in the study population; *OR – odds ratio for increase of per minor allele

^†^Marginal Association Test *P*-value: p-value based on test of SNP main effect assuming additive effect

^‡^HT Interaction *P*-value: *P*-value based on 2df Joint test for interaction with HT; ^‡^HT Interaction *FDR*: *FDR* based on 2df Joint test for interaction with HT; ^‡^E-alone Interaction *P*-value: *P*-value based on test for interaction with E-alone; ^‡^E+P Interaction *P*-value: *P*-value based on test for interaction with E+P; ^‡^rank: rank of SNPs based on HT interaction test with 2df
