# Supplementary material for: Exploring the interaction between SNP genotype and postmenopausal hormone therapy effects on stroke risk
Source: Genome Med. 2012 Jul 13;4(7):57. doi: 10.1186/gm358 (PMC3580413; doi:10.1186/gm358)
Supplement: Additional file 2 — Stroke odds ratio for E-alone and E+P, by genotype of SNPs in the F13A1 region in women of European ancestry. This file presents analyses corresponding to Table 3, with cases and controls restricted to be of European ancestry. [file gm358-S2.DOC]

**Additional File 2.** Stroke odds ratio for E-alone and E+P, by genotype of SNPs in *F13A1 region* in women of European ancestry.

1. rs2154299.

|  |  | | | **SNP genotype** | | | | | | | | | |  | |  | |
| --- | --- | --- | --- | --- | --- | --- | --- | --- | --- | --- | --- | --- | --- | --- | --- | --- | --- |
| **E-alone** | |  |  | | **GG** |  |  | **GA** |  |  | **AA** |  |  | |  | |  |
|  | | Number of Cases | **OR^†^** | | 95% | CI | **OR^†^** | 95% | CI | **OR^†^** | 95% | CI | p-2df* | | p-1df^#^ | |  |
| All | | 261 | **1.509** | | (1.144, | 1.991) | **1.067** | (0.602, | 1.89) | **0.511** | (0.094, | 2.791) | 0.27365 | | 0.11728 | |  |
| Ischemic | | 193 | **1.675** | | (1.208, | 2.321) | **1.214** | (0.624, | 2.361) | **0.682** | (0.114, | 4.079) | 0.45829 | | 0.21904 | |  |
| Hemorrhagic | | 36 | **1.095** | | (0.529, | 2.269) | **0.409** | (0.079, | 2.108) | **NA** | (NA, | NA) | 0.26325 | | 0.26325 | |  |
| **E+P** | |  |  | |  |  |  |  |  |  |  |  |  | |  | |  |
| All | | 374 | **1.522** | | (1.196, | 1.938) | **0.714** | (0.472, | 1.082) | **0.238** | (0.027, | 2.13) | 0.00195 | | 0.00043 | |  |
| Ischemic | | 268 | **1.583** | | (1.187, | 2.112) | **0.702** | (0.431, | 1.144) | **0.238** | (0.027, | 2.13) | 0.00426 | | 0.00098 | |  |
| Hemorrhagic | | 65 | **1.072** | | (0.618, | 1.857) | **0.714** | (0.248, | 2.059) | **NA** | (NA, | NA) | 0.50334 | | 0.50334 | |  |

(b) rs12194855.

|  |  | | | **SNP genotype** | | | | | | | | | |  | |  | |
| --- | --- | --- | --- | --- | --- | --- | --- | --- | --- | --- | --- | --- | --- | --- | --- | --- | --- |
| **E-alone** | |  |  | | **AA** |  |  | **AG** |  |  | **GG** |  |  | |  | |  |
|  | | Number of Cases | **OR^†^** | | 95% | CI | **OR^†^** | 95% | CI | **OR^†^** | 95% | CI | p-2df* | | p-1df^#^ | |  |
| All | | 261 | **1.509** | | (1.144, | 1.991) | **1.022** | (0.581, | 1.800) | **0.682** | (0.114, | 4.079) | 0.35128 | | 0.14806 | |  |
| Ischemic | | 193 | **1.675** | | (1.208, | 2.321) | **1.214** | (0.624, | 2.361) | **0.682** | (0.114, | 4.079) | 0.45829 | | 0.21904 | |  |
| Hemorrhagic | | 36 | **1.095** | | (0.529, | 2.269) | **0.409** | (0.079, | 2.108) | **NA** | (NA, | NA) | 0.26325 | | 0.26325 | |  |
| **E+P** | |  |  | |  |  |  |  |  |  |  |  |  | |  | |  |
| All | | 374 | **1.504** | | (1.181, | 1.917) | **0.751** | (0.499, | 1.131) | **0.238** | (0.027, | 2.13) | 0.00389 | | 0.00094 | |  |
| Ischemic | | 268 | **1.557** | | (1.166, | 2.080) | **0.752** | (0.466, | 1.214) | **0.238** | (0.027, | 2.13) | 0.00882 | | 0.00225 | |  |
| Hemorrhagic | | 65 | **1.072** | | (0.618, | 1.857) | **0.714** | (0.248, | 2.059) | **NA** | (NA, | NA) | 0.50334 | | 0.50334 | |  |

^†^OR: estimated intervention odds ratio

*p-2df: p-value regression randomization assignment on indicator for one or two minor alleles

^#^p-1df: p-value regressing randomization assignment on number of minor alleles

NA indicates information (data) not available
