# Supplementary material for: Exploring the interaction between SNP genotype and postmenopausal hormone therapy effects on stroke risk
Source: Genome Med. 2012 Jul 13;4(7):57. doi: 10.1186/gm358 (PMC3580413; doi:10.1186/gm358)
Supplement: Additional file 3 — Stroke odds ratio for E-alone and E+P, by genotype of SNPs in PCSK9 region for women with European ancestry. This file presents analyses corresponding to Table 4, with cases and controls restricted to be of European ancestry. [file gm358-S3.DOC]

**Additional File 3.** Stroke odds ratio for E-alone and E+P, by genotype of SNPs in *PCSK9 region* for women with European ancestry.

1. rs630431

|  |  | | | | **SNP genotype** | | | | | | | | | | | | | | | | |  | | |  | | |  | |
| --- | --- | --- | --- | --- | --- | --- | --- | --- | --- | --- | --- | --- | --- | --- | --- | --- | --- | --- | --- | --- | --- | --- | --- | --- | --- | --- | --- | --- | --- |
| **E-alone** | |  |  | | | | **AA** |  | |  | | **AG** | |  | |  | | | **GG** |  | | |  | | |  | | |  |
|  | | Number of Cases | | **OR^†^** | | 95% | | | CI | | **OR^†^** | | 95% | | CI | | **OR^†^** | 95% | | | CI | | | p-2df* | | | p-1df^#^ | | |
| All | | 261 | | **1.762** | | (1.23, | | | 2.524) | | **1.08** | | (0.742, | | 1.573) | | 1.208 | (0.541, | | | 2.697) | | | 0.17029 | | | 0.11378 | | |
| Ischemic | | 193 | | **2.077** | | (1.36, | | | 3.171) | | **1.315** | | (0.845, | | 2.045) | | 0.613 | (0.223, | | | 1.688) | | | 0.0555 | | | 0.01794 | | |
| Hemorrhagic | | 36 | | **0.795** | | (0.296, | | | 2.135) | | **0.818** | | (0.323, | | 2.072) | | ∞ | --- | | |  | | | 0.20859 | | | 0.36077 | | |
| **E+P** | |  | |  | |  | | |  | |  | |  | |  | |  |  | | |  | | |  | | |  | | |
| All | | 374 | | **0.953** | | (0.718, | | | 1.264) | | **1.633** | | (1.174, | | 2.27) | | 1.645 | (0.783, | | | 3.457) | | | 0.03628 | | | 0.01769 | | |
| Ischemic | | 268 | | **0.953** | | (0.679, | | | 1.336) | | **1.626** | | (1.106, | | 2.391) | | 1.786 | (0.757, | | | 4.212) | | | 0.08294 | | | 0.03456 | | |
| Hemorrhagic | | 65 | | **0.649** | | (0.337, | | | 1.252) | | **1.786** | | (0.757, | | 4.212) | | 1.429 | (0.239, | | | 8.551) | | | 0.15859 | | | 0.09382 | | |

1. rs568052

|  |  | | | **SNP genotype** | | | | | | | | | |  | |  | |
| --- | --- | --- | --- | --- | --- | --- | --- | --- | --- | --- | --- | --- | --- | --- | --- | --- | --- |
| **E-alone** | |  |  | | **AA** |  |  | **AG** |  |  | **GG** |  |  | |  | |  |
|  | | Number of Cases | **OR^†^** | | 95% | CI | **OR^†^** | 95% | CI | **OR^†^** | 95% | CI | p-2df* | | p-1df^#^ | |  |
| All | | 261 | **1.778** | | (1.237, | 2.555) | **1.079** | (0.744, | 1.566) | **1.208** | (0.541, | 2.697) | 0.1582 | | 0.10666 | |  |
| Ischemic | | 193 | **2.111** | | (1.375, | 3.241) | **1.306** | (0.845, | 2.021) | **0.613** | (0.223, | 1.688) | 0.0497 | | 0.01554 | |  |
| Hemorrhagic | | 36 | **0.795** | | (0.296, | 2.135) | **0.818** | (0.323, | 2.072) | **∞** | --- |  | 0.20859 | | 0.36077 | |  |
| **E+P** | |  |  | |  |  |  |  |  |  |  |  |  | |  | |  |
| All | | 374 | **0.973** | | (0.733, | 1.291) | **1.588** | (1.143, | 2.205) | **1.645** | (0.783, | 3.457) | 0.06068 | | 0.0274 | |  |
| Ischemic | | 268 | **0.981** | | (0.699, | 1.377) | **1.565** | (1.066, | 2.296) | **1.786** | (0.757, | 4.212) | 0.13684 | | 0.05501 | |  |
| Hemorrhagic | | 65 | **0.649** | | (0.337, | 1.252) | **1.786** | (0.757, | 4.212) | **1.429** | (0.239, | 8.551) | 0.15859 | | 0.09382 | |  |

^†^OR: estimated intervention odds ratio

*p-2df: p-value regression randomization assignment on indicator for one or two minor alleles

^#^p-1df: p-value regressing randomization assignment on number of minor alleles
